# Dual-target lymphatic embolization for refractory chylous ascites after nephroureterectomy

**DOI:** 10.1093/bjrcr/uaag038

**Published:** 2026-08-25

**Authors:** Ryoma Kobayashi, Kohei Hamamoto, Emiko Chiba, Soichiro Kojima, Tatsuya Ogawa, Hiroyuki Fujii, Mitsuru Matsuki, Masayuki Kurokawa, Toru Sugihara, Satoshi Ando, Tetsuya Fujimura, Harushi Mori

**Affiliations:** Department of Radiology, Jichi Medical University, Shimotsuke-City, Tochigi 329-0498, Japan; Department of Radiology, Jichi Medical University, Shimotsuke-City, Tochigi 329-0498, Japan; Department of Radiology, Jichi Medical University, Shimotsuke-City, Tochigi 329-0498, Japan; Department of Radiology, Jichi Medical University, Shimotsuke-City, Tochigi 329-0498, Japan; Department of Radiology, Jichi Medical University, Shimotsuke-City, Tochigi 329-0498, Japan; Department of Radiology, Jichi Medical University, Shimotsuke-City, Tochigi 329-0498, Japan; Department of Radiology, Jichi Medical University, Shimotsuke-City, Tochigi 329-0498, Japan; Department of Urology, Jichi Medical University, Shimotsuke-City, Tochigi 329-0498, Japan; Department of Urology, Jichi Medical University, Shimotsuke-City, Tochigi 329-0498, Japan; Department of Urology, Jichi Medical University, Shimotsuke-City, Tochigi 329-0498, Japan; Department of Urology, Jichi Medical University, Shimotsuke-City, Tochigi 329-0498, Japan; Department of Radiology, Jichi Medical University, Shimotsuke-City, Tochigi 329-0498, Japan

**Keywords:** chylous ascites, percutaneous lymphatic embolization, nephroureterectomy, lymphadenectomy

## Abstract

We report a case of refractory chylous ascites following nephroureterectomy and para-aortic lymphadenectomy for renal pelvic carcinoma, which was successfully treated with percutaneous lymphatic embolization. An 83-year-old man underwent nephroureterectomy with para-aortic lymph node dissection for renal pelvic carcinoma. Postoperatively, >1000 mL/day of chylous ascites was continuously drained. Despite conservative management, high-output ascites persisted. Transnodal lymphangiography via the inguinal lymph nodes and post-lymphangiographic CT revealed lymphatic leakage at the nephroureterectomy site. Percutaneous lymphatic embolization was planned as a minimally invasive procedure. Under CT guidance, embolization of a lymphopseudoaneurysm and para-aortic lymph nodes near the leakage site was successfully performed using N-butyl cyanoacrylate. Ascitic fluid output decreased significantly postoperatively, and the drainage tube was removed the following day. This case highlights the importance of post-lymphangiographic CT in identifying actionable lymphatic targets and demonstrates that an anatomically tailored dual-target embolization strategy can achieve effective sealing when central duct access is not feasible.

## Introduction

Refractory chylous ascites is an uncommon but clinically significant complication following retroperitoneal urological surgery. The reported incidence is approximately 5%-6% after nephrectomy and 1%-2% after para-aortic lymph node dissection.^1^^,^^2^ Although many cases respond to conservative management, persistent high-output chylous ascites may lead to malnutrition, immunosuppression, and prolonged hospitalization. First-line treatment typically consists of dietary modification, total parenteral nutrition (TPN), and administration of somatostatin analogues, with reported success rates of 60%-80%.^2^^,^^3^ However, high-output leaks (>1000 mL/day) are more likely to require interventional or surgical treatment.^2^^,^^3^

Recently, image-guided lymphatic interventions have emerged as minimally invasive alternatives to surgical ligation.^4–8^ Reported techniques include transnodal lymphangiography, percutaneous glue embolization using N-butyl cyanoacrylate (NBCA), and retrograde transvenous thoracic duct embolization.^7^^,^^9^ However, optimal embolization strategies remain under discussion.

We report a case of persistent high-output chylous ascites successfully treated using a structured dual-target embolization strategy.

## Case presentation and imaging findings

**Figure 1:**
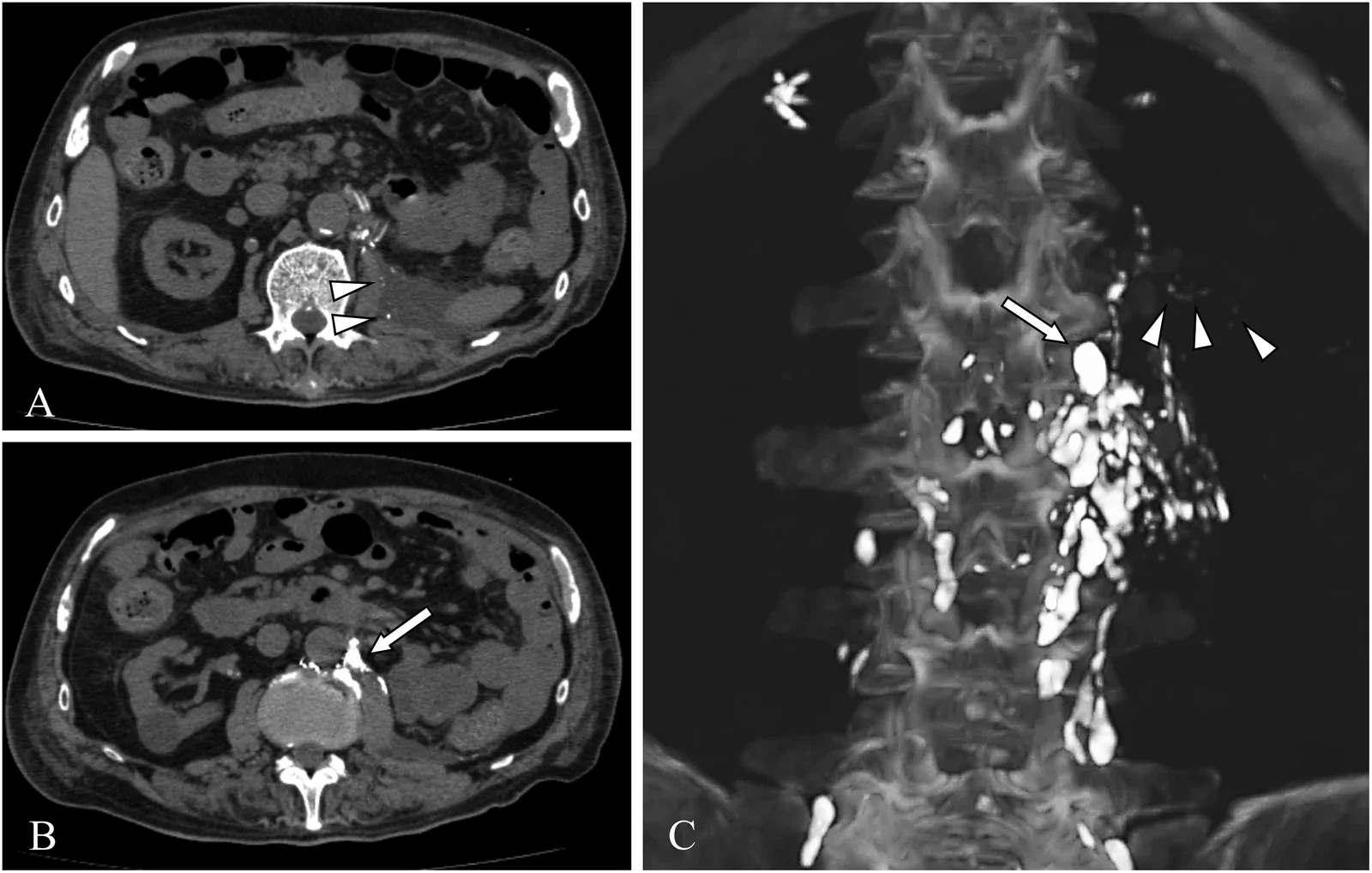
Noncontrast CT performed the day after transnodal lymphangiography. (A) Deposition of ethiodized oil in the retroperitoneal fluid collection near the nephrectomy site (arrowheads). (B) A lymphopseudoaneurysm is observed in the para-aortic region adjacent to the surgical site (arrow). (C) Maximum intensity projection image in the coronal view (bone window) shows a lymphopseudoaneurysm (arrow) and the leakage site (arrowheads).

An 83-year-old man under follow-up in our urology department had been diagnosed with left renal pelvic carcinoma 18 months earlier. Although nephroureterectomy was initially planned, he declined surgery and underwent local laser ablation. Follow-up CT later demonstrated tumor regrowth in the renal pelvis, and surgical resection was reconsidered. His medical history included robot-assisted radical prostatectomy for prostate cancer and coronary artery stent placement for ischemic heart disease. He was taking low-dose aspirin, which was temporarily discontinued perioperatively. Preoperative laboratory tests revealed anemia (hemoglobin 8.4 g/dL) and renal dysfunction (serum creatinine 3.39 mg/dL), without other significant abnormalities. The clinical stage was T3N1M0 (stage IV). Although systemic chemotherapy was considered the standard treatment option at this stage, the patient declined chemotherapy and strongly preferred surgical management. After thorough multidisciplinary discussion and comprehensive informed consent, robot-assisted left nephroureterectomy with para-aortic lymph node dissection was performed. Postoperatively, more than 1000 mL/day of milky ascitic fluid consistent with chylous ascites was continuously drained from the pelvic drain. Conservative management included dietary modification with a low-fat diet supplemented with medium-chain triglycerides, TPN, and continuous intravenous administration of a somatostatin analog. Despite these conservative treatments, high-output drainage persisted. On postoperative day (POD) 11, transnodal lymphangiography via a 12-mm left inguinal lymph node was performed using ethiodized oil (Lipiodol Ultra Fluid; Guerbet Japan). No leakage was identified fluoroscopically during the procedure. However, CT obtained the following day demonstrated accumulation of ethiodized oil in the retroperitoneal space near the nephrectomy bed (Figure 1A), consistent with postoperative lymphorrhea. In addition, a para-aortic lymphopseudoaneurysm adjacent to the surgical site was identified (Figure 1B and C), representing a localized lymphatic extravasation cavity formed by disruption of lymphatic vessels.^4^ Conservative management was continued in anticipation of a possible therapeutic effect from Lipiodol; however, because of persistent high-volume drainage, a multidisciplinary discussion was conducted, and percutaneous lymphatic embolization was planned as a minimally invasive alternative to surgical reintervention. Written informed consent was obtained.

## Treatment

**Figure 2:**
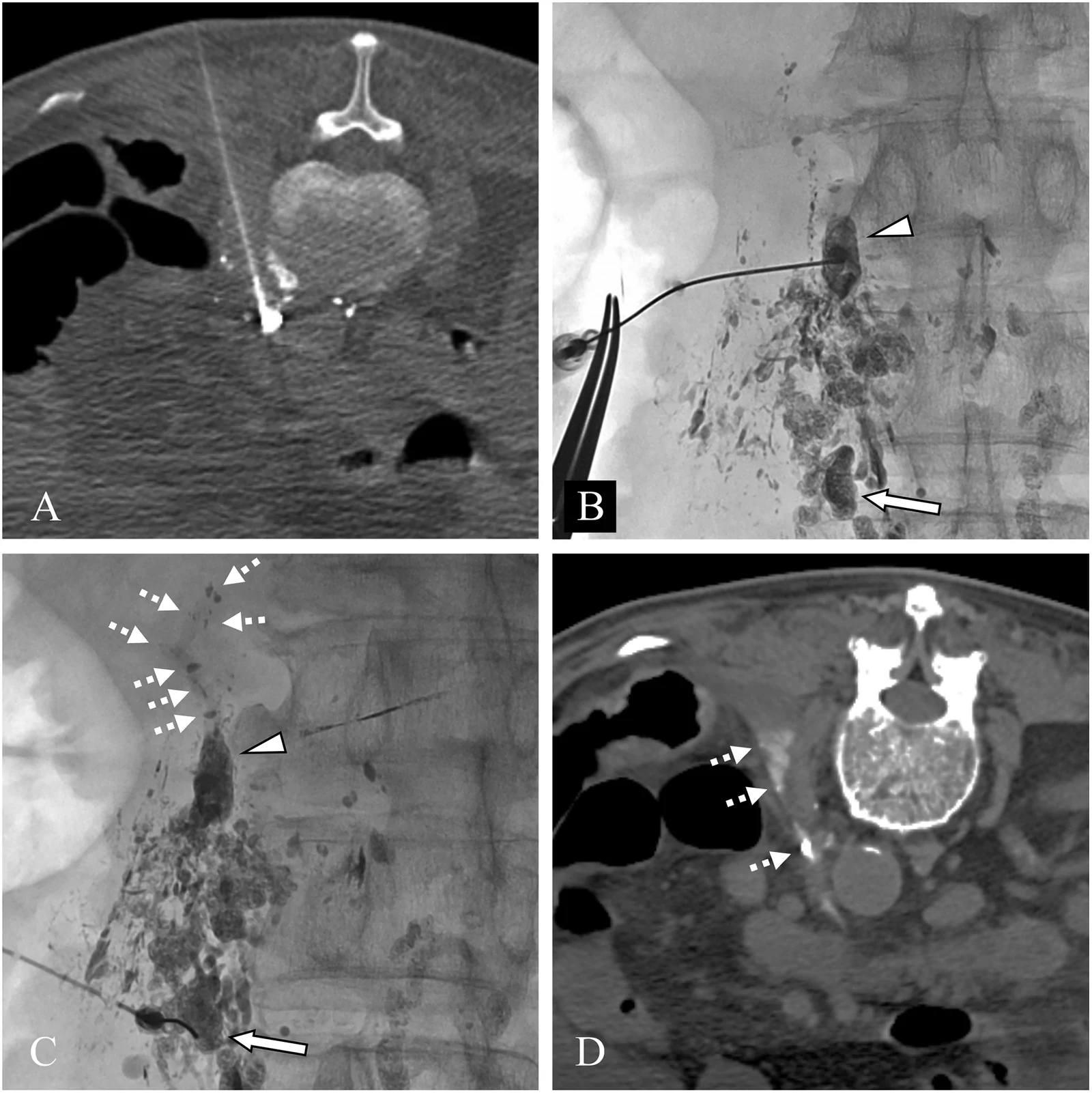
Percutaneous lymphatic embolization. (A) CT-guided puncture of the lymphopseudoaneurysm. (B) Fluoroscopy-guided injection of an NBCA/ethiodized oil mixture for embolization. Notably, the lymphopseudoaneurysm is filled with the NBCA/ethiodized oil mixture (arrowhead). The arrow indicates the caudal para-aortic lymph node. Note that the left and right sides are reversed because the image was acquired in the prone position. (C) CT-guided embolization of the caudal para-aortic lymph node (arrow) using the NBCA/ethiodized oil mixture. Fluoroscopy shows ethiodized oil distribution in the target lymph node, lymphatic pathway, and retroperitoneal space (dashed arrows), appearing as faint hyperattenuating areas consistent with extravasated iodinated contrast. The arrowhead indicates the embolized lymphopseudoaneurysm. Note that the left and right sides are reversed because the image was acquired in the prone position. (D) Post-injection CT shows distribution of ethiodized oil corresponding to the leakage site (arrows).

On POD 18, percutaneous lymphatic embolization was performed. Repeat lymphangiography via a 10-mm left inguinal lymph node was successfully performed in the supine position; however, the central lymphatic structures, including the cisterna chyli and thoracic duct, were not visualized because the lymphatic pathways had been disrupted by lymph node dissection. Therefore, a targeted embolization strategy was adopted. First, the lymphopseudoaneurysm adjacent to the nephrectomy bed was punctured in the prone position under CT fluoroscopic guidance using a 21-gauge PTCD needle (Figure 2A). Aspiration of ethiodized oil confirmed correct intralesional positioning. Although contrast injection did not demonstrate extravasation into the retroperitoneum, the lymphopseudoaneurysm was located cranial to the presumed site of lymphatic disruption and was therefore considered part of the leakage pathway. A total of 1.5 mL of a 33.3% NBCA/ethiodized oil mixture was injected to achieve occlusion (Figure 2B). Subsequently, a caudally located para-aortic lymph node supplying lymphatic inflow to the leakage site was punctured using the same technique. Contrast injection at this site demonstrated leakage into the retroperitoneal space, confirming active communication with the lymphatic disruption. Two milliliters of a 20% NBCA/ethiodized oil mixture were slowly injected under fluoroscopic monitoring (Figure 2C). Post-injection CT confirmed distribution of the embolic material corresponding to the suspected lymphatic leakage route (Figure 2D), and the procedure was completed. No intra- or postoperative complications occurred.

## Outcome and follow-up

**Figure 3:**
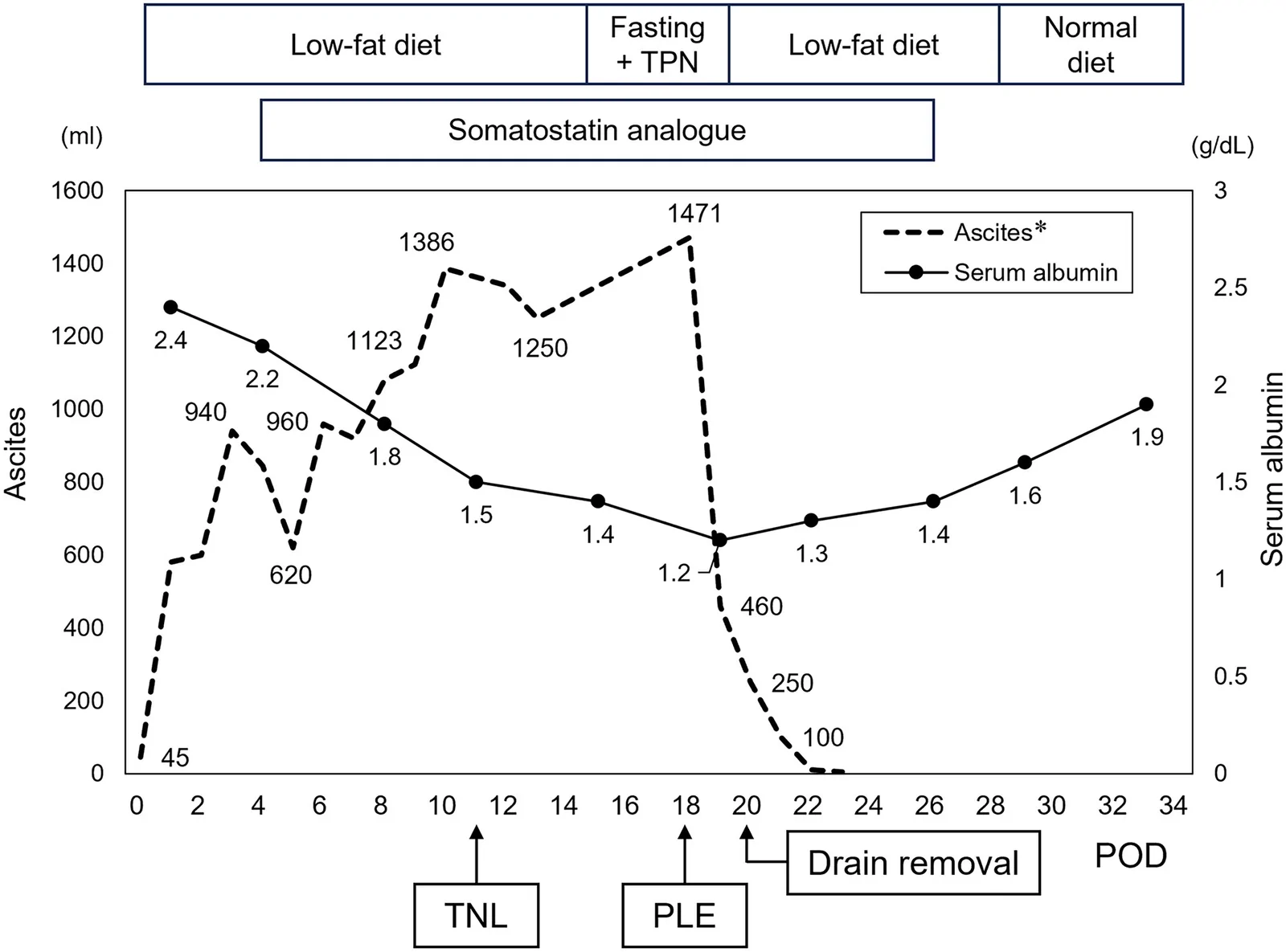
Summary of the clinical course. Abbreviations: PLE, percutaneous lymphatic embolization; POD, postoperative day; TNL, transnodal lymphangiography; TPN, total parenteral nutrition. * The asterisk indicates the sum of the drain output and fluid leakage from the drain puncture site.

Drain output decreased markedly the following day, and the drain was subsequently removed (Figure 3). Somatostatin analog therapy was discontinued 7 days after embolization. The patient resumed a fat-containing diet without recurrence of ascites. At 3-month follow-up, there was no clinical or imaging evidence of recurrent chylous ascites.

## Discussion

Postoperative chylous ascites represents a challenging complication in retroperitoneal surgery, particularly when high-output leakage persists despite conservative therapy.^1^^,^^2^ Although dietary restriction and somatostatin analogues achieve resolution in a substantial proportion of low-output cases, persistent drainage exceeding 1000 mL/day is associated with low spontaneous closure rates and prolonged hospitalization.^2^^,^^3^ In this clinical context, early interventional management may shorten recovery and prevent metabolic deterioration.

Lymphatic embolization has emerged as a minimally invasive alternative to surgical ligation, with reported technical success rates of 80%-100% and clinical success rates of 70%-90%.^4–8^ Compared with surgical reintervention, percutaneous embolization offers lower morbidity and the ability to target anatomically complex leaks. The key to successful lymphatic embolization lies in achieving complete occlusion of the disrupted lymphatic segment. In general, this is accomplished by embolizing both the inflow and outflow channels of the injured lymphatic segment or by directly targeting the leakage point itself. Most previously reported cases have therefore focused on embolization of a single structure, such as the thoracic duct, cisterna chyli, or a leaking lymphatic channel.

However, when direct catheterization of the injured lymphatic vessel or embolization of the leakage point is technically challenging, NBCA embolization of the lymphatic inflow alone can be performed.^7^^,^^8^ In such situations, incomplete interruption of the disrupted lymphatic pathway may allow persistent leakage. In our case, central lymphatic structures, including the cisterna chyli and thoracic duct, were not visualized, making transvenous or central access impractical. Although embolization of lymphopseudoaneurysms has been reported as an effective strategy,^4^ its efficacy may depend on the anatomical location of the lesion and its relationship to the true leakage site. Indeed, contrast extravasation was not reproduced during embolization of the lymphopseudoaneurysm in the present case, raising concern that embolization of the lymphopseudoaneurysm alone might be insufficient. Therefore, a structured dual-target embolization strategy was adopted. First, the cranial lymphopseudoaneurysm was embolized to occlude the outflow side of the leakage pathway. Second, the caudal para-aortic lymph nodes demonstrating active extravasation were embolized to interrupt lymphatic inflow. This combined approach achieved rapid cessation of drainage and durable resolution of ascites. To our knowledge, a deliberately structured dual-target strategy combining embolization of the cranial lymphopseudoaneurysm and interruption of lymphatic inflow through the caudal para-aortic lymph nodes in a single session has rarely been detailed in postoperative chylous ascites following nephroureterectomy.

A higher NBCA concentration (33.3%) was selected for embolization of the cranial lymphopseudoaneurysm to promote rapid polymerization and minimize reflux into surrounding tissues. Because NBCA polymerizes more slowly in lymphatic fluid than in blood, a higher concentration was necessary to achieve prompt solidification and secure local occlusion. Conversely, a lower concentration (20%) was used for embolization of the caudal para-aortic lymph nodes to allow controlled penetration into the lymphatic inflow channels while reducing the risk of excessive cranial migration. As the cranial lymphopseudoaneurysm had already been occluded, the risk of unintended cranial migration beyond the target was limited, thereby facilitating more efficient sealing of the leakage pathway. Tailoring polymerization kinetics to the anatomical target may enhance both procedural efficacy and safety, particularly when lymphatic flow dynamics are difficult to predict. Although foreign-body granuloma formation after NBCA use has rarely been reported in other clinical settings,^10^ we found no reports of granuloma formation associated with extralymphatic NBCA deposition after embolization for postoperative lymphatic leakage. Nevertheless, this potential delayed complication should be considered during follow-up.

Alternative approaches include retrograde transvenous thoracic duct embolization and surgical ligation. Retrograde access requires visualization and catheterization of the thoracic duct via the venous angle,^9^ which was not feasible in this case. Surgical reintervention remains effective but is associated with increased morbidity, particularly in elderly patients with cardiovascular comorbidities, as in our patient. In such scenarios, image-guided glue embolization offers a physiologically targeted and organ-preserving solution.

This case highlights the importance of post-lymphangiographic CT in delineating the cranial lymphopseudoaneurysm and caudal lymphatic inflow pathways. Careful anatomical assessment enabled selection of dual embolization targets and tailored adjustment of NBCA concentration to optimize procedural control and safety.

In conclusion, a structured dual-site NBCA embolization strategy achieved rapid and durable resolution of refractory postoperative chylous ascites. This approach may offer an effective minimally invasive option for complex lymphatic leaks when conventional central lymphatic access is not feasible.

## Learning points

- Persistent high-output chylous ascites after retroperitoneal surgery may require image-guided intervention when conservative therapy fails.
- Delayed CT following transnodal lymphangiography can identify lymphatic leakage even when fluoroscopy is negative.
- Postoperative lymphopseudoaneurysm may represent a focal lymphatic disruption amenable to direct puncture.
- Dual-target NBCA embolization of both the outflow and inflow sides of the lymphatic leakage pathway can effectively control refractory lymphorrhea.
- Targeted percutaneous lymphatic embolization provides a minimally invasive alternative to surgical re-exploration.
